# Chemical Composition, Antimicrobial Activity, and Antioxidant Activity of *Ocotea minarum* (Nees & Mart.) Mez.

**DOI:** 10.1155/2019/5736919

**Published:** 2019-04-28

**Authors:** Allan Belarmino Rodrigues, Adriana Araújo de Almeida-Apolonio, Tamaeh Monteiro Alfredo, Fabiana Gomes da Silva Dantas, Jaqueline Ferreira Campos, Claudia Andrea Lima Cardoso, Kely de Picoli Souza, Kelly Mari Pires de Oliveira

**Affiliations:** ^1^Faculty of Exact Sciences and Technology, Federal University of Grande Dourados, Dourados, MS 79804-970, Brazil; ^2^Faculty of Medicine, Federal University of Mato Grosso do Sul, Campo Grande, MS 79070-900, Brazil; ^3^Faculty of Health Sciences, Federal University of Grande Dourados, Dourados, MS 79804-970, Brazil; ^4^Faculty of Biological and Environmental Science, Federal University of Grande Dourados, Dourados, MS 79804-970, Brazil; ^5^Course of Chemistry, State University of Mato Grosso do Sul, Dourados, MS 79804-970, Brazil

## Abstract

*Ocotea minarum* is a native plant from Brazil, popularly known as “canelinha” or “canela vassoura.” The objective of this study was to investigate the chemical composition of the extracts of the bark and the leaves of *O. minarum* and to evaluate its antimicrobial and antioxidant activities. The phenolic compounds, flavonoids and tanins, were quantified with the reagents Folin-Ciocalteu, aluminium chloride, and vanillin. The chemical profile was performed by HPLC-DAD. The minimum inhibitory concentration was evaluated by the microdilution in a broth method. The antioxidant activity was measured by the capture of free radicals 2,2-diphenyl-1-picrylhydrazyl and 2,2′-azinobis(3-ethylbenzothiazoline-6-sulfonic acid). In addition, protection against oxidative hemolysis and generation of malondialdehyde were evaluated in human erythrocytes. The composition of the extracts included the caffeic acid, p-coumaric acid, and rosmarinic acid, besides the flavonoids quercetin and luteolin. The EEL showed bacteriostatic action of 1000 *μ*g/mL for all evaluated *Salmonella* Typhimurium, *Salmonella* Enteritidis, *Pseudomonas aeruginosa*, and *Proteus mirabilis*, and the EHEB had a moderate antifungal action against *Candida krusei* and *Cryptococcus gattii* (250 *μ*g/mL). IC_50_ values of 8.19 (EEL) and 4.51 *μ*g/mL (EEB) in the assay with DPPH and 6.25 (EEL) and 2.87 *μ*g/mL (EEB) in the assay with ABTS were obtained. Up to the 3rd hour of oxidative hemolysis testing induced by AAPH, the EEB and EEL had a protective action, reducing the malondialdehyde. In conclusion, the data indicate that the *O. minarum* extracts can be evaluated as bioactive supplies for the development of new drugs for the prevention and treatment of diseases related to oxidative stress and microbial infections.

## 1. Introduction

The use of medicinal plants as raw material to develop products for therapeutic purposes is common. It has been estimated that from the 60,000 species discovered, the biological potential of 800 species has been evaluated [1, 2]. It is known that plants with secondary metabolites, such as alkaloids, coumarins, tannins, terpenes, and flavonoids, are sources of several bioactive compounds that are beneficial to human health [3].

These compounds are described as having various biological activities, including antimicrobial action. Microbial infections are growing at an alarming level [4, 5] owing to the ability of bacteria and yeasts to acquire and transmit genetic resistance material, leading to the constant evolution of multidrug-resistant microorganisms that threaten public health [6–8]. In this milieu, medicinal plants are receiving special attention for the development of new antimicrobial agents due to their therapeutic activity, low toxicity, and economic viability when compared with those of traditional allopathic compounds [9, 10].

Another problem that affects public health is the degeneration of tissues and biomolecules caused by the imbalance of oxidative substances and antioxidants in the body, leading to oxidative stress. Oxidative stress is an important factor that triggers cardiovascular and neurodegenerative diseases, inflammation, diabetes, and premature aging, among others [11, 12]. An approach to reduce this condition is the use of antioxidant substances that can act in the neutralization or even in the capture of reactive species, thus, minimizing oxidative tissue damage [13, 14].

Compounds such as butylated hydroxyanisole (BHA) and butylated hydroxytoluene (BHT) are the widely used synthetic antioxidants, but are questioned due to their nutritional value and possible mutagenic, carcinogenic, and toxic effects, including hepatotoxicity [15–17]. Generally, natural antioxidants have low toxicity and are easily biodegradable, making them an effective alternative to reduce oxidative stress [18, 19].

Additionally, tropical and subtropical plant species are of increasing research interest, as they have diverse properties, such as potent antimicrobial and antioxidant activities [13, 20]. In this context, the members of the genus *Ocotea* stand out, which are among the most expressive of the family Lauraceae. Among these, approximately 300–400 species are found in the tropical and subtropical regions [21, 22], and it has been estimated that 120–160 species are found in Brazil [23] dispersed in the biomes of Atlantic Forest and Cerrado [24]. Studies on species of the genus *Ocotea* have demonstrated several biological activities, such as antimicrobial [25–29], antioxidant [25, 26, 29–32], anti-inflammatory [30, 33], and antiprotozoal activities [34].

Among the genus *Ocotea*, the species *Ocotea minarum* (Nees & Mart) Mez., a medium-sized tree, popularly known as “canelinha” or “canela vassoura” [35], contains aporphine alkaloids [36], indole alkaloids, coumarins, flavonoids, biflavonoids, bis-lignans, alkyl benzene, steroids, sesquiterpenes, nor-sesquiterpenes, and terpene lactone [37]. However, studies on the antibacterial and antioxidant properties of *O. minarum* are limited. Thus, the objective of the present study was to (1) determine the phytochemical profile, (2) evaluate the antifungal activity, and (3) for the first time, evaluate the antibacterial activity and antioxidant potential of *O. minarum* bark and leaf extracts.

## 2. Materials and Methods

### 2.1. Plant Material

The stem bark and leaves of *O. minarum* were collected from the Mata dos Macacos farm (22°08′47.2^″^S, 54°54′54.1^″^W) in the city of Dourados, Mato Grosso do Sul, Brazil. The samples were identified by Prof. Dr. Zefa Valdivia Pereira and deposited in the Herbarium of the Faculty of Biological and Environmental Sciences (FCBA) in the Federal University of Grande Dourados (UFGD) under the registered number 5275 DDMS.

### 2.2. Preparation of Extracts

The barks (246.80 g) and leaves (237.28 g) were sterilized and dried in a laminar flow cabinet at 40°C for 10 and 6 days, respectively. Subsequently, the samples were pulverized using a cutting mill. The sample was extracted with 800 mL of 80% ethyl alcohol at ambient temperature with regular shaking at every 24 h. After 72 h, the mixture was filtered using a filter paper (0.45 *μ*m), rotoevaporated at 35°C and lyophilized to obtain the ethanolic extract of barks (EEB) and leaves (EEL) of *O. minarum*.

Solvents with increasing polarity (hexane and ethyl acetate) were used to prepare the liquid-liquid partitions to obtain hydroalcoholic extract (HYEB and HYEF), hexane extract (HEEB and HEEL), and ethyl acetate extract (EAEB and EAEL) from the barks and leaves of *O. minarum*, respectively [38].

### 2.3. Determination of Phenolic Compounds

The content of phenolic compounds was determined as described by Djeridane et al. [39]. To obtain 100 *μ*L of the EEB and EEL at a concentration of 100 *μ*g/mL, 500 *μ*L of Folin-Ciocalteu reagent and 1 mL of distilled water were added and incubated at ambient temperature for 1 min. Subsequently, 1.5 mL of 20% sodium carbonate was added to this solution and incubated for 2 h in the dark at ambient temperature. The absorbance was read at 760 nm. The concentration of phenolic compounds was calculated by preparing an analytical curve using gallic acid as standard at concentrations of 100, 200, 300, 400, 500, 600, 700, 800, 900, and 1000 *μ*g/mL. With the data, the linear regression was developed and the equation of line was obtained, with a linear correlation coefficient of *R*^2^ = 0.997, slope *a* = −0.018, and linear coefficient *b* = 0.0016. The results are expressed as milligrams of gallic acid equivalent (mg/GAE) per gram of extract. All analyses were performed in triplicate.

### 2.4. Determination of Flavonoids

The content of flavonoids was determined by the method described by Lin and Tang [40]. Five hundred microliters of EEB and EEL at a concentration of 100 *μ*g/mL was added to 1.5 mL of 95% ethyl alcohol, 0.1 mL of 10% aluminum chloride hexahydrate (AlCl_3_), 0.1 mL of 1 M potassium acetate (CH_3_COOK), and 2.8 mL of deionized water. The reaction mixture was incubated at ambient temperature for 40 min, and the absorbance was read using a spectrophotometer at a wavelength of 415 nm. To determine the concentration of flavonoids, an analytical curve was prepared using quercetin as standard at concentrations of 10, 20, 30, 40, and 50 *μ*g/mL, and the respective absorbance was read. With the obtained data, the linear regression and the equation of the line with *R*^2^ = 0.999, *a* = 0.0019, and *b* = 0.0105 were obtained. The results are expressed as milligrams of quercetin equivalent (mg/QE) per gram of extract. All analyses were performed in triplicate.

### 2.5. Determination of Tannins

The tannins were quantified by the vanillin reaction according to the method of Broadhurst and Jones [41], adapted by Agostini-Costa et al. [42]. Five milliliters of freshly prepared vanillin reagent (vanillin-HCL-methanol, 4 : 10 : 86) was taken in a test tube and 1 mL of EEB or EEL, at a concentration of 100 *μ*g/mL, was added and agitated for 30 s. The reaction mixture was undisturbed for 15 min, and the absorbance was read at 490 nm. The quantification was done using an external calibration curve with catechin as standard, at concentrations of 2.5, 5, 10, 20, 30, and 40 *μ*g/mL, and their absorbance was read, to obtain the equation of line with *R*^2^ = 0.998, *a* = 0.006, and *b* = 0.0018. The results are expressed as milligrams of catechin equivalent (mg/CAE) per gram of extract. All analyses were performed in triplicate.

### 2.6. High-Performance Liquid Chromatography with Diode-Array Detector (DAD)

The extracts were analyzed by high-performance liquid chromatography (HPLC) using a Shimadzu model, equipped with the C18 Phenomenex Gemini column (25 cm × 4.6 mm × 5 *μ*m). The flow rate was 1 mL/min, with scans between 200 and 800 nm, and the injection volume was 10 *μ*L. The mobile phase was as follows: 6% acetic acid in water, 2 mM sodium acetate (eluent A), and acetonitrile (eluent B). The gradient used was as follows: 0 min 5% B, 45 min 15% B, 55 min 30% B, 60 min 50% B, and 65 min 100% B [43].

### 2.7. Antimicrobial Activity

#### 2.7.1. Microorganisms for Antimicrobial Testing

The microorganisms were obtained from the *American Type Culture Collection* (ATCC, Rockville, MD, USA). The microorganisms included the following yeasts: *Candida albicans* (90028), *Candida glabrata* (2001), *Candida krusei* (6558), *Candida tropicalis* (750), *Candida parapsilosis* (22019), *Candida dubliniensis* (MYA-646), *Cryptococcus gattii* (56990), *Cryptococcus neoformans* (32045), *Rhodotorula glutinis* (2527), and *Rhodotorula mucilaginosa* (64684). Gram-positive bacteria included *Staphylococcus aureus* (25923), *Staphylococcus epidermidis* (12228), and *Bacillus cereus* (11778). Gram-negative bacteria included *Salmonella* Typhimurium (14028), *Salmonella* Enteritidis (13076), *Pseudomonas aeruginosa* (27853), and *Proteus mirabilis* (35659).

To prepare the inoculum, the microorganisms were suspended in sterile saline solution (0.9% NaCl) and adjusted using a spectrophotometer (Quimis, Diadema, SP, Brazil). 90% (±2) transmittance at 530 nm corresponding to a suspension of 2.5 × 10^6^ UFC/mL concentration, for yeasts, and an absorbance of 0.110 (± 0.005) at 625 nm corresponding to a concentration of 1.5 × 10^8^ UFC/mL, for bacteria, were used.

#### 2.7.2. Determination of Minimum Inhibitory Concentration (MIC) of the Extract

The susceptibility assay was performed by the broth microdilution technique, according to the guidelines of the Clinical and Laboratory Standards Institute. The standards of document M27-A3 [44] and document M07-A9 [45] with some modifications for the use of plant extracts were used for yeasts and bacteria, respectively.

The EEB, EEL, HYEB, HEEB, EAEB, HYEF, HEEL, and EAEL were dissolved in dimethylsulfoxide (DMSO) and then diluted (1 : 2) to concentrations of 1.9 and 1000 *μ*g/mL. As a control, 100 *μ*L of culture medium and 50 *μ*L of extracts were added to the wells of column 1 of a 96-well microplate. The amount of inoculum added to the plates was 10 and 100 *μ*L for bacteria and yeasts, respectively. The culture medium used was RPMI-1640 for yeasts and Müller Hinton broth for bacteria. The microplates were incubated at 35°C for 48 h for *Candida* sp. and 72 h for *Cryptococcus* sp. and *Rhodotorula* sp. and at 37°C for 24 h for the bacteria. Fluconazole was used as a control antifungal agent and ampicillin as a control antibiotic. The experiments were performed in triplicate at three different times.

#### 2.7.3. Minimal Fungicidal Concentration (MFC) and Minimal Bactericidal Concentration (MBC)

Aliquots from a microdilution plate (of MIC assay) were transferred to a Petri dish containing Sabouraud Dextrose agar or Müller Hinton agar to the fungicidal and bactericidal activities, respectively. The plates were incubated at 35°C for 48 h for *Candida* sp. and 72 h for *Cryptococcus* sp. and *Rhodotorula* sp. and at 37°C for 24 h for bacteria. The MFC and MBC were defined as the lowest concentration capable of inhibiting colony growth [46].

### 2.8. Antioxidant Activity

#### 2.8.1. Capture Test of 2,2-Diphenyl-1-picrylhydrazyl (DPPH) Radical

The antioxidant activity of EEB and EEL was evaluated by the method of Sharma et al. [47] using the free radical 2,2-diphenyl-1-picrylhydrazyl (DPPH). Ethanolic solution of DPPH (0.11 mM, 1800 *μ*L) was incubated with 200 *μ*L of the positive controls, ascorbic acid, and hydroxybutyltoluene (BHT) or EEB and EEL at concentrations of 0.1-2000 *μ*g/mL in 80% ethanol. After 30 min of incubation at ambient temperature (protected from light), the absorbance was read using a spectrophotometer (Quimis, Diadema, SP, Brazil) at 517 nm; the absorbance of each sample was transformed into free radical inhibition percent using formula (1). Three independent experiments were performed in triplicate.

(1) $$\begin{array}{c} \text{Radical scavenging }\left(\%\right)=\frac{\left(\text{Abs control}-\text{Abs sample}\right)}{\text{Abs control}}\times 100. \end{array}$$

#### 2.8.2. Capture of Free Radical 2,2′-Azinobis(3-ethylbenzothiazoline-6-sulfonic Acid) (ABTS)

The free radical inhibition capacity of the EEB and EEL was evaluated according to the method described by Re et al. [48] using 2,2′-azinobis(3-ethylbenzothiazoline-6-sulfonic acid) (ABTS). Ascorbic acid and BHT were used as positive controls. The stock solution of ABTS was prepared using potassium persulfate at 12–16 h prior to the experiment and maintained at ambient temperature, protected from light. The ABTS radical was diluted in ethanol PA until the absorbance reached 0.70 ± 0.05 at 734 nm and was incubated for 6 min with the extracts or controls at the concentrations used in the antioxidant assay. The volume of ABTS and extract used was 1980 *μ*L and 20 *μ*L, respectively. The absorbance was read using a spectrophotometer (Quimis, Diadema, SP, Brazil) at 734 nm. The results were expressed as % of free radical inhibition using formula (2). Three independent experiments were performed in triplicate.

(2) $$\begin{array}{c} \text{Percent inibition of ABTS }\left(\%\right)=\frac{\left(\text{Abs control}-\text{Abs sample}\right)}{\text{Abs controle}}\times 100. \end{array}$$

#### 2.8.3. Hemolysis Oxidative Test Induced by 2,2′-Azobis(2-amidinopropane) (AAPH)

The protection effect of EEB and EEL against lipid peroxidation was evaluated by the method of oxidative hemolysis induced by 2,2′-azobis(2-amidinopropane) dihydrochloride (AAPH, Sigma-Aldrich), described by Campos et al. [49]. After the approval of the Research Ethics Committee (REC) of the Federal University of Grande Dourados (UFGD) with the process number 5160, 25 mL of blood was collected from a single, healthy, nonsmoking adult individual and stored in tubes containing the anticoagulant sodium citrate. From this sample, a 10% solution of red blood cells was prepared using 0.9% NaCl solution.

The extracts (final concentrations of 10-125 *μ*g/mL) were preincubated with the erythrocyte solution at 37°C for 30 min. After this period, 0.9% NaCl solution or 50 mM AAPH solution was added to investigate whether the extracts promote hemolysis in erythrocytes or inhibit oxidative hemolysis, respectively. The samples were incubated in a water bath at 37°C for 4 h, and the mixture was homogenized steadily during this period. After 120, 180, and 240 min, the samples were removed, centrifuged at 1500 rpm/10 min, and then, the absorbance of the supernatant using a spectrophotometer (Quimis, Diadema, SP, Brazil) at 540 nm was read. The positive control ascorbic acid was maintained under the same conditions in assays. As a solvent control, the erythrocytes were incubated with ethanol at a final concentration of 0.4%. Three independent experiments were performed in duplicates. The percent of hemolysis was determined using formula 3, where the absorbance of total hemolysis represents erythrocytes incubated with distilled water.

(3) $$\begin{array}{c} \text{Hemolysis }\left(\%\right)=\frac{\text{Abs sample }}{\text{Mean Abs total hemolysis}}\times 100. \end{array}$$

#### 2.8.4. Determination of Malondialdehyde (MDA)

A byproduct of lipid peroxidation, MDA, was measured to assess the ability of extracts to protect erythrocytes against oxidative stress. For this, the ascorbic acid or extracts (final concentrations of 10-125 *μ*g/mL) were preincubated with erythrocyte suspension at 37°C for 30 min. After this period, the oxidizing agent AAPH, at 50 mM concentration, was added and the mixture was incubated for 4 h, under periodic agitation. After 120, 180, and 240 min, the samples were centrifuged at 1500 rpm for 10 min and 500 *μ*L of the remaining supernatant was added to 1 mL of 10 nM thiobarbituric acid (TBA; Merck), solubilized in monosodium potassium phosphate buffer (75 mM) at pH 2.5. The control was prepared by the mixture of TBA and 20 mM MDA solution. The samples were incubated in a water bath at 96°C for 45 min.

Subsequently, the reaction mixture was placed on an ice bath for 15 min to cease the reaction, and then, 4 mL of butanol was added to the mixture, with subsequent vortexing followed by centrifugation at 3000 rpm for 5 min, according to the method described by Campos et al. [50]. The absorbance of the supernatant was recorded using a spectrophotometer (Quimis, Diadema, SP, Brazil) at 532 nm. The content of MDA is expressed as nmol/mL, obtained using

(4) $$\begin{array}{c} \text{MDA}\left(\text{nmol}/\text{mL}\right)=\frac{\text{Abs sample }\left(20\times 220.32\right)}{\text{Abs control}}. \end{array}$$

### 2.9. Statistical Analyses

The results are presented as the mean ± standard error of the mean. The analysis of variance (ANOVA) followed by Dunnett's test was performed for the comparison between more than two experimental groups. The comparison between two experimental groups was performed by the Newman-Keuls test. The statistical analyses were performed using the statistical program GraphPad Prism 5 (GraphPad Software Inc., USA). The data were considered significant when the *P* value was <0.05.

## 3. Results

### 3.1. Chemical Composition

The content of phenolic compounds and tannins in the EEB was higher than those in the EEL. The level of flavonoids in the EEL was higher than that in the EEB (Table 1).

**Figure 1:**
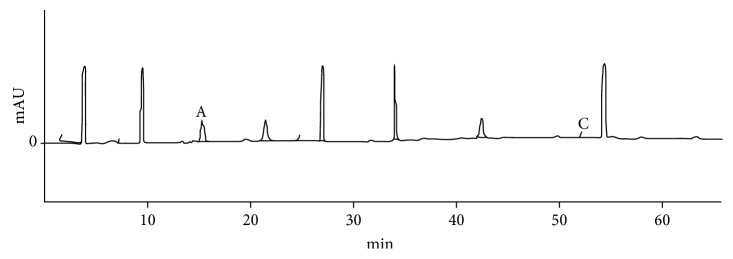
High-performance liquid chromatography of the EEB of *Ocotea minarum*. A caffeic acid (15 min). C rosmarinic acid (53.7 min).

**Figure 2:**
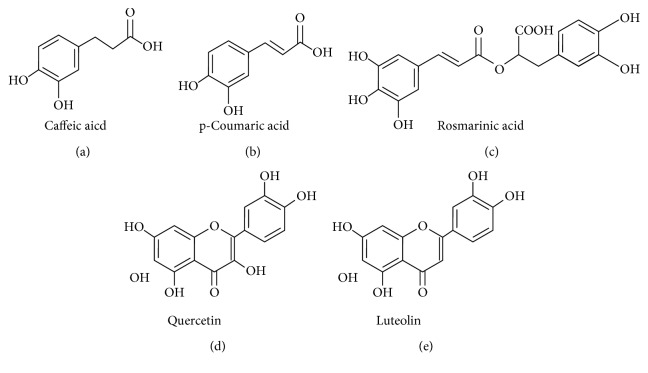
Compounds identified by high-performance liquid chromatography of the ethanolic extract from bark (EEB) and leaves (EEL) of *Ocotea minarum*.

**Figure 3:**
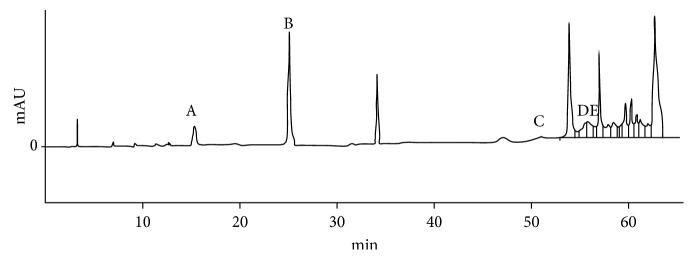
High-performance liquid chromatography of the EEL of *Ocotea minarum*. A caffeic acid (15.1 min). B p-coumaric acid (25.2 min). C rosmarinic acid (53.7 min). D quercetin (59.1 min). E luteolin (59.7 min).

The compounds were identified by HPLC and by the analysis of ultraviolet spectra of each peak. Caffeic acid (15 min) and rosmarinic acid (53.7 min) were identified in the EEB (Figures 1 and 2). In the EEL, caffeic acid (15.1 min), p-coumaric acid (25.2 min), rosmarinic acid (53.7 min), quercetin (59.1 min), and luteolin (59.7 min) were identified (Figures 2 and 3).

### 3.2. Antimicrobial Activity

We evaluated the antimicrobial activity of different extracts against yeasts, gram-positive bacteria, and gram-negative bacteria (Tables 2 and 3). The HEEB was effective against all evaluated yeast strains, except against *R. glutinis* and *R. mucilaginosa*. The EEL, HYEL, and HEEB exhibited fungistatic and fungicidal action against *C. krusei* (Table 2).

The HYEB presented bacteriostatic and bactericidal action against the evaluated gram-positive bacteria. The EEL at a concentration of 1000 *μ*g/mL showed antibacterial activity against all the evaluated gram-negative bacteria (Table 3).

### 3.3. Antioxidant Activity

#### 3.3.1. Antioxidant Activity of Extracts by the DPPH and ABTS Assays

The results are expressed as the IC_50_ value and maximal activity of both the extracts (Table 4). The EEB had IC_50_ results similar to those of the antioxidant control, ascorbic acid, in both assays. Additionally, it presented superior results compared to the synthetic antioxidant BHT, requiring concentrations 1.3 and 2.2 times smaller to inhibit 50% of DPPH and ABTS free radicals, respectively. The EEL had IC_50_ results similar to those of the BHT in both assays; however, it showed less activity than the standard antioxidant ascorbic acid.

#### 3.3.2. Hemolytic Activity

**Figure 4:**
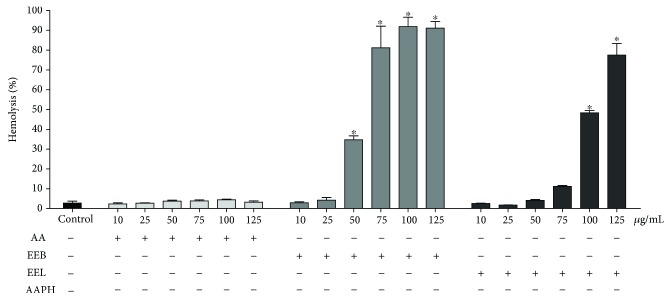
Hemolysis evaluation in human erythrocytes incubated for 4 h with the antioxidant standard ascorbic acid (AA) and ethanolic extract of bark (EEB) and leaves (EEL) of *O. minarum* at concentrations of 10-125 *μ*g/mL. ^∗^*P* > 0.05 vs. control.

After 4 h of incubation (Figure 4), ascorbic acid did not induce hemolysis at the evaluated concentrations. The EEB and EEL showed a pattern similar to that of the control ascorbic acid, not inducing hemolysis at lower concentrations. However, they induced hemolysis at concentrations of >25 *μ*g/mL in EEB and >100 *μ*g/mL in EEL.

#### 3.3.3. Protection against Oxidative Hemolysis

**Figure 5:**
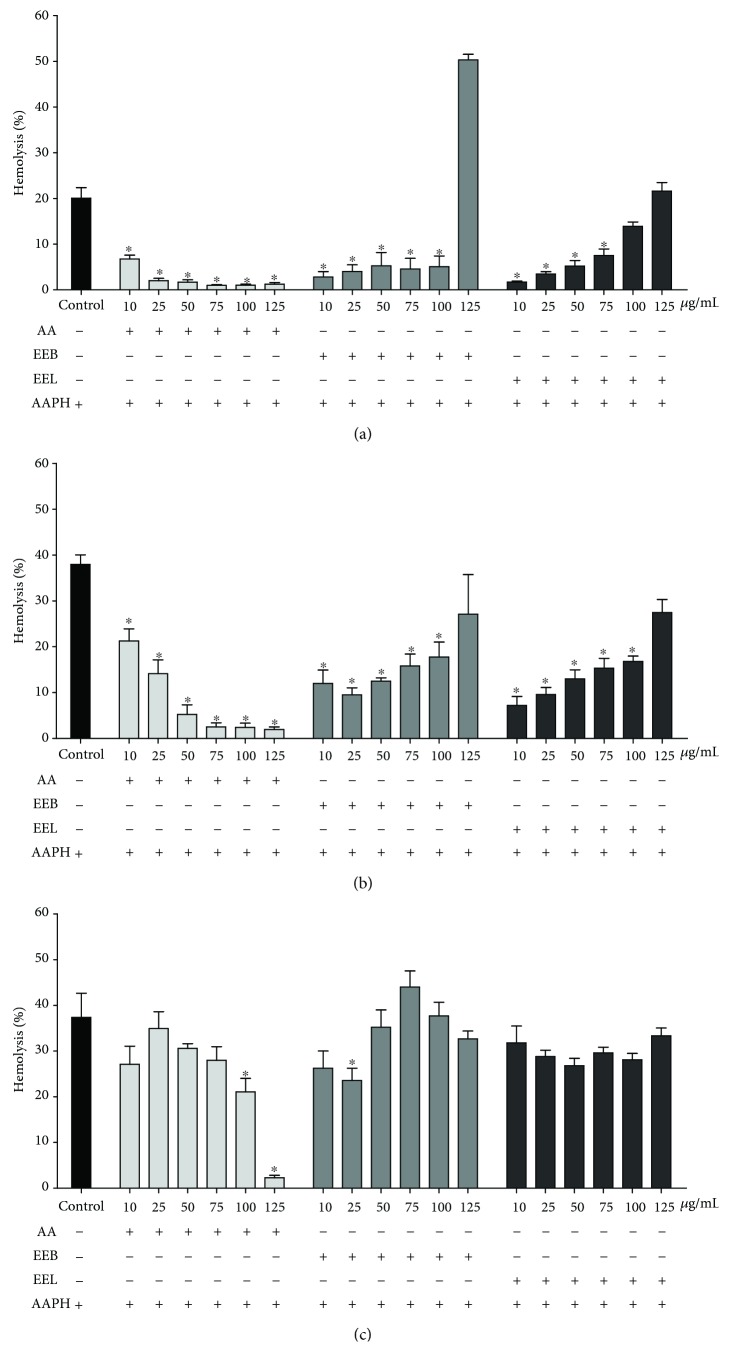
Assessment of hemolysis in human erythrocytes incubated for (a) 120, (b) 180, and (c) 240 min with the oxidizing agent AAPH with different concentrations of ascorbic acid, EEB, and EEL (10–125 *μ*g/mL). ^∗^*P* > 0.05 vs. the AAPH control group.

After assessing the hemolytic activity of extracts in human erythrocytes, the protective activity of EEB and EEL was observed in the erythrocytes subjected to oxidative hemolysis by AAPH (Figure 5). At 120 and 180 min (Figures 5(a) and 5(b)), the extracts exhibited protective action against oxidative hemolysis at the lowest concentrations of 10 to 100 *μ*g/mL. At 240 min, only the EEB at a concentration of 25 *μ*g/mL maintained this activity compared with that of the AAPH control (Figure 5(c)).

#### 3.3.4. Generation of Malondialdehyde

**Figure 6:**
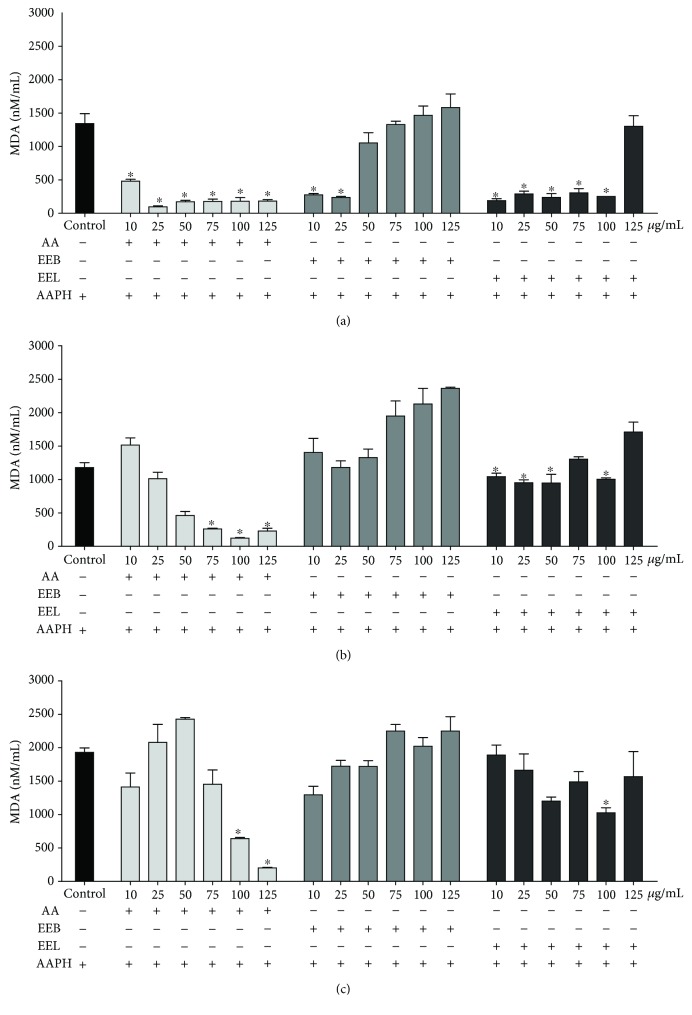
Concentration of malondialdehyde (MDA) in human erythrocytes incubated for (a) 120, (b) 180, and (c) 240 min with the oxidizing agent AAPH with different concentrations of ascorbic acid, EEB, and EEL (10–125 *μ*g/mL). ^∗^*P* > 0.05 vs. the AAPH control group.

The capacity of the extracts to protect erythrocytes incubated with the oxidizing agent AAPH against lipid peroxidation was assessed by the generation of MDA. At 120 min, the erythrocytes incubated with EEB at concentrations of 10 and 25 *μ*g/mL showed a reduction in the generation of MDA by approximately 83%, when compared with that of the AAPH control group (Figure 6(a)). When incubated with the EEL at concentrations of 10 to 100 *μ*g/mL, there was a reduction in the formation of MDA by approximately 85%.

In addition, at 180 min (Figure 6(b)), only the EEL reduced the formation of MDA by approximately 40% at concentrations of 10 to 100 *μ*g/mL and, at 240 min, reduced in 47% the formation of MDA, at a concentration of 100 *μ*g/mL, which was significantly different from that of the AAPH group (Figure 6(c)).

## 4. Discussion

Medicinal plants are sources of important biomolecules, including phenolic compounds, which are described as having various biological activities as antibacterial, antifungal, anti-inflammatory, antioxidant, and antitumor. The phenolic compounds caffeic acid and p-coumaric acid, identified by HPLC in the extracts of *O. minarum,* have already been related to antioxidant and antimicrobial activities in plants [51, 52].

The extracts of *O. minarum* inhibited the growth of 15 out of seventeen microorganisms evaluated by the broth microdilution assay. Microorganisms have several defense mechanisms; gram-positive cells have only one lipopolysaccharide-phospholipid layer, thus conferring a higher penetration sensitivity of antibiotic substances, whereas gram-negative cells have a double layer of lipopolysaccharide-phospholipid, hindering the passage of molecules into the bacterial cell [53]. There are also mechanisms for the efflux of small molecules, inactivation of enzymes, and loss of porins in the plasma membrane [54, 55]. Furthermore, some yeasts, such as *Cryptococcus* sp. and *Rhodotorula* sp., are encapsulated, which confer them greater protection [56].

According to Morales et al. [57] different parameters of antimicrobial activity were considered: MIC < 100 *μ*g/mL, active; MIC 100–500 *μ*g/mL, moderate; MIC 500–1000 *μ*g/mL, weak antimicrobial activity; and MIC > 1000 *μ*g/mL, inactive. Based on the adopted criteria, the EHEB showed good antifungal activity against *C. neoformans* and moderate activity against *C. tropicalis*, *C. gattii*, and *C. krusei*. *Cryptococcus neoformans* might have gained resistance to fluconazole, and *C. krusei* is intrinsically resistant to the same antifungal agent and is often isolated from opportunistic and invasive infections caused by *Candida* species [58]. Therefore, a search for new techniques for the development of new antimicrobials is important to improve the treatment of infections. With respect to gram-positive bacteria, in the present study, the EAEL and HYEB showed good antibacterial activity against *Bacillus cereus* and moderate activity against *S. aureus* and *S. epidermidis*.

Bruni et al. [29] showed the antibacterial activity of the essential oil of *Ocotea quixos* flowers against *S. aureus* and *E. faecalis*. Gil et al. [27] demonstrated that the essential oil of *Ocotea caudata* leaves has antibacterial action against *B. subtilis* and *S. aureus*. In the present study, the EEB and EEL of *O. minarum* did not present active antibacterial action against gram-negative bacteria. This suggests that the extracts probably act on the cell wall and peptidoglycan bonds of gram-positive bacteria and do not have good activity against the gram-negative bacteria due to the presence of a double layer of lipopolysaccharide-phospholipid, conferring less sensitivity.

The antimicrobial activity exhibited by the EEB and EEL of *O. minarum* is probably due to the presence of sesquiterpenes: lanceolic acid, curcumen-12-oic acid, (+)-E-exo-abergamoten-12-oico acid, loliolide terpene lactone, and nor-sesquiterpene [37]. The flavonoids quercetin and luteolin, present in EEB and EEL, have been associated with antimicrobial activity, as they can penetrate cellular phospholipid membranes due to their hydrophobicity [59, 60]. To the best of our knowledge, the present study is the first to report the antibacterial activity of *O. minarum*.

Further, the phenolic acids caffeic acid and p-coumaric acid and the flavonoid quercetin identified are also associated with antioxidant activity, due to their direct action in the capture of free radicals [61–63]. The EEB and EEL exhibited promising antioxidant activity in the DPPH and ABTS assay, when compared with that of ascorbic and BHT, reference antioxidants. In particular, EEB presented a higher antioxidant potential than EEL in both the tests.

Other authors describe the antioxidant activity of species of the genus *Ocotea* [25, 29]. Comparatively, in the present study, the EEB and EEL of *O. minarum* showed a better antioxidant potential than other species of the same genus.

The redox balance of the body is regulated by endogenous and exogenous mechanisms, in which the excess of free radicals is a fundamental factor causing numerous diseases, such as diabetes, atherosclerosis, cardiovascular diseases, cancer, and premature aging, among others [11]. Therefore, the search for natural antioxidants has increased in order to replace the synthetic compounds that might pose harmful effects on human health [64].

When evaluating the antioxidant properties of extracts in human erythrocytes, although the EEB and EEL at the highest concentrations induced high hemolysis rates, both promoted the reduction of AAPH-induced lipid peroxidation at lower doses and during the early evaluation times, protecting the erythrocytes from cell lysis. The peroxidation of the phospholipid membrane is induced by reactive species, especially oxygen and nitrogen species, causing cell lysis and death. Furthermore, a byproduct of this reaction is malondialdehyde [65, 66], which is a toxic product leading to the release of unsaturated fatty acids and the fragmentation of phospholipids, causing mutations and cellular rupture [19]. The EEB and EEL reduced the generation of malondialdehyde during the early evaluation times, contributing to the maintenance of a cellular structure, at low concentrations. Thus, protection against this type of cellular damage is considered a positive effect [11], as it is associated with the reduction of damage caused by oxidative stress and its complications.

Luteolin, a flavonoid present in the extract of the leaves of *O. minarum*, has been described by modulating the expression of antioxidant enzymes superoxide dismutase and catalase, besides reducing the levels of malondialdehyde generated in an oxidative stress model *in vivo* [67]. The flavonoid quercetin and the polyphenol rosmarinic acid, also identified in extracts of the *O. minarum*, have already been described by activation of the transcriptional factor Nrf-2 (nuclear factor erythroid 2), an important cell antioxidant mechanism [68, 69].

In conclusion, the data indicate that the *O. minarum* extracts can be evaluated as bioactive supplies for the development of new drugs for the prevention and treatment of diseases related to microbial infections and oxidative stress.

## Figures and Tables

**Table 1:** Content of phenolic compounds, flavonoids, and condensed tannins in the EEB and EEL of *O*. *minarum*.

Sample	Phenolic compounds	Flavonoids	Condensed tannins
	(mg GAE/g)	(mg QE/g)	(mg CAE/g)
EEB	156.4 ± 3.1	72.1 ± 1.6	16.1 ± 0.2
EEL	134.5 ± 2.3	78.5 ± 1.4	12.9 ± 0.1

**Table 2:** Minimum inhibitory concentration (MIC) and minimal fungicide concentration (MFC) of the bark and leaf extracts of *O. minarum* against yeasts (*μ*g/mL).

Microorganism	EEB		EAEB		HEEB		HYEB		EEL		EAEL		HEEL		HYEL		FLU
	MIC	MFC	MIC	MFC	MIC	MFC	MIC	MFC	MIC	MFC	MIC	MFC	MIC	MFC	MIC	MFC	MIC
*Candida albicans*	>1000	>1000	>1000	>1000	1000	>1000	>1000	>1000	1000	>1000	>1000	>1000	>1000	>1000	>1000	>1000	2
*Candida glabrata*	>1000	>1000	>1000	>1000	500	500	>1000	>1000	500	>1000	500	500	>1000	>1000	>1000	>1000	8
*Candida krusei*	500	>1000	250	>1000	250	250	250	>1000	250	500	125	>1000	>1000	>1000	125	250	32
*Candida tropicalis*	500	1000	1000	>1000	250	250	1000	>1000	>1000	>1000	500	>1000	>1000	>1000	1000	1000	1
*Candida parapsilosis*	250	>1000	1000	>1000	500	500	>1000	>1000	250	>1000	500	>1000	>1000	>1000	>1000	>1000	2
*Candida dubliniensis*	500	1000	250	>1000	1000	>1000	>1000	>1000	250	1000	500	500	>1000	>1000	>1000	>1000	0.25
*Cryptococcus gattii*	500	>1000	1000	>1000	250	>1000	>1000	>1000	500	>1000	250	>1000	>1000	>1000	>1000	>1000	8
*Cryptococcus neoformans*	500	>1000	500	>1000	62.5	>1000	>1000	>1000	500	>1000	125	>1000	>1000	>1000	>1000	>1000	8
*Rhodotorula glutinis*	>1000	>1000	>1000	>1000	>1000	>1000	>1000	>1000	>1000	>1000	>1000	>1000	>1000	>1000	>1000	>1000	32
*Rhodotorula mucilaginosa*	>1000	>1000	>1000	>1000	>1000	>1000	>1000	>1000	>1000	>1000	>1000	>1000	>1000	>1000	>1000	>1000	32

EEB: ethanolic extract of bark; EAEB: ethyl acetate extract of bark; HEEB: hexane extract of bark; HYEB: hydroalcoholic extract of bark; EEL: ethanolic extract of leaf; EAEL: ethyl acetate extract of leaf; HEEL: hexane extract of leaf; HYEF: hydroalcoholic extract of leaf; FLU: fluconazole. Biological analysis was performed in duplicate with three independent experiments.

**Table 3:** Minimum inhibitory concentration (MIC) and minimal bactericidal concentration (MBC) of the bark and leaf extracts of *O. minarum* against gram-positive and gram-negative bacteria (*μ*g/mL).

Microorganism	EEB		EAEB		HEEB		HYEB		EEL		EAEL		HEEL		HYEL		AMP
	MIC	MBC	MIC	MBC	MIC	MBC	MIC	MBC	MIC	MBC	MIC	MBC	MIC	MBC	MIC	MBC	MIC
*Staphylococcus aureus*	500	>1000	250	>1000	500	>1000	125	1000	500	>1000	125	500	1000	>1000	>1000	>1000	32
*Staphylococcus epidermidis*	1000	>1000	250	>1000	1000	>1000	125	1000	1000	>1000	250	>1000	>1000	>1000	500	>1000	64
*Bacillus cereus*	500	500	500	>1000	500	>1000	62.5	500	250	>1000	62.5	250	1000	>1000	125	1000	32
*Salmonella* Typhimurium	>1000	>1000	>1000	>1000	1000	>1000	>1000	>1000	1000	>1000	1000	>1000	>1000	>1000	>1000	>1000	32
*Salmonella* Enteritidis	1000	>1000	>1000	>1000	1000	>1000	1000	>1000	1000	>1000	1000	>1000	1000	>1000	1000	>1000	32
*Pseudomonas aeruginosa*	>1000	>1000	1000	>1000	500	>1000	1000	>1000	1000	>1000	>1000	>1000	1000	>1000	1000	>1000	—
*Proteus mirabilis*	>1000	>1000	1000	>1000	>1000	>1000	1000	>1000	1000	>1000	1000	>1000	>1000	>1000	>1000	>1000	32

EEB: ethanolic extract of bark; EAEB: ethyl acetate extract of bark; HEEB: hexane extract of bark; HYEB: hydroalcoholic extract of bark; EEL: ethanolic extract of leaf; EAEL: ethyl acetate extract of leaf; HEEL: hexane extract of leaf; HYEL: hydroalcoholic extract of leaf; AMP: ampicillin. Biological analysis was performed in duplicate with three independent experiments. —: not tested against this microorganism.

**Table 4:** Antioxidant activity of free radical capture DPPH and ABTS of extracts EEB and EEL of *O. minarum* and from the standard antioxidants: ascorbic acid (AA) and BHT.

Samples	DPPH			ABTS		
	IC_50_ (*μ*g/mL)	Maximal activity		IC_50_ (*μ*g/mL)	Maximal activity	
		*μ*g/mL	%		*μ*g/mL	%
AA	4.36 ± 1.63	10	95.58	1.16 ± 0.14	5	99.61
BHT	6.07 ± 0.79	50	86.13	6.42 ± 0.43	50	97.88
EEB	4.51 ± 0.49	10	83.93	2.87 ± 0.42	10	94.32
EEL	8.19 ± 1.25	50	90.12	6.25 ± 0.89	50	95.59

IC_50_, required concentration to capture 50% of the free radicals in the reaction. Values are the means ± standard error of the mean (SEM).

## Data Availability

The chemical composition, antimicrobial, and antioxidant data used to support the findings of this study are included within the article.
